# Predicting Changes in Cardiovascular Risk Factors in Type 2 Diabetes in the Post-UKPDS Era: Longitudinal Analysis of the Swedish National Diabetes Register

**DOI:** 10.1155/2013/241347

**Published:** 2013-02-28

**Authors:** Aliasghar Ahmad Kiadaliri, Philip M. Clarke, Ulf-G. Gerdtham, Peter Nilsson, Björn Eliasson, Soffia Gudbjörnsdottir, Katarina Steen Carlsson

**Affiliations:** ^1^Division of Health Economics, Department of Clinical Sciences, Malmö University Hospital, Lund University, 20502 Malmö, Sweden; ^2^Health Economics & Management, Institute of Economic Research, Lund University, 22007 Lund, Sweden; ^3^Department of Health Management and Economics, School of Public Health, Tehran University of Medical Sciences, Tehran 141556447, Iran; ^4^School of Public Health, University of Sydney, Sydney, NSW 2006, Australia; ^5^Department of Economics, Lund University, 22363 Lund, Sweden; ^6^Department of Clinical Sciences, Malmö University Hospital, Lund University, 20502 Malmö, Sweden; ^7^Department of Medicine, Sahlgrenska University Hospital, University of Gothenburg, 41345 Göteborg, Sweden

## Abstract

The aim of the current study was to provide updated time-path equations for risk factors of type-2-diabetes-related cardiovascular complications for application in risk calculators and health economic models. Observational data from the Swedish National Diabetes Register were analysed using Generalized Method of Moments estimation for dynamic panel models (*N* = 5,043, aged 25–70 years at diagnosis in 2001–2004). Validation was performed using persons diagnosed in 2005 (*n* = 414). Results were compared with the UKPDS outcome model. The value of the risk factor in the previous year was the main predictor of the current value of the risk factor. People with high (low) values of risk factor in the year of diagnosis experienced a decreasing (increasing) trend over time. BMI was associated with elevations in all risk factors, while older age at diagnosis and being female generally corresponded to lower levels of risk factors. Updated time-path equations predicted risk factors more precisely than UKPDS outcome model equations in a Swedish population. Findings indicate new time paths for cardiovascular risk factors in the post-UKPDS era. The validation analysis confirmed the importance of updating the equations as new data become available; otherwise, the results of health economic analyses may be biased.

## 1. Introduction

Type 2 diabetes is well known as a risk factor for cardiovascular disease (CVD) [1, 2], with a risk 2–4 times higher for patients with diabetes compared with the general population [3, 4]. The risk of death is doubled for persons with type 2 diabetes [5], and more than 75% of mortality among patients with type 2 diabetes is attributed to CVD [6]. Some of the main risk factors for increased risk of CVD deaths in patients with type 2 diabetes include level of serum lipids, hyperglycaemia, systolic blood pressure (BP), smoking, and obesity [7–11]. One or more of these risk factors have also been shown to play a role in predicting the occurrence of other types of complications including amputation, renal impairment, and eye-related complications [12, 13]. These risk factors are commonly used in clinical risk equations [14, 15] as well as health economic simulation models [16]. 

There is increasing interest in forecasting outcomes for people with diabetes, based on demographic and clinical characteristics. For example, it is recommended that the intensity of treatment for diabetes should be determined in part by the level of cardiovascular risk [17]. More generally, risk calculators and outcome tables [18] have been developed to inform patients with a given set of clinical characteristics of their risk of events and other outcomes such as life expectancy. Risk modelling is also used by health economists when quantifying the benefits of new technologies and interventions as well as the reductions in the cost of complications through better management of the disease. A key aspect of risk modelling is the understanding of how risk factors change over time, since these changes influence the progression of the disease and the risk of complications. In the context of type 2 diabetes, the UKPDS outcome model [13] is the main source of predicting changes in risk factors over time and is widely used in health economic simulation models. One problem with using this model is that the levels and trends in common risk factors may be different across regions and over time owing to changes in clinical practice patterns, demographic characteristics of patients, and potentially other factors such as lifestyle [19]. For example, two recent studies have shown that mean BMI and systolic BP have substantially changed worldwide since 1980 [20, 21], implying that estimations based on data from 1980 may not reflect the current situation. These differences should be taken into account when making risk predictions.

Studying the change in risk factors over time requires longitudinal data which are not readily available in many countries. The initiation of the Swedish National Diabetes Register (NDR) in 1996, including data on different risk factors, has provided the opportunity to examine the change in risk factors in people with diabetes in routine clinical practice over time. 

In this study, the time path during 2001–2008 for five cardiovascular risk factors (HbA1c, systolic BP, BMI, total to HDL cholesterol ratio (TC : HDL), and LDL cholesterol) was predicted for persons newly diagnosed with type 2 diabetes in 2001–2004. Changes in risk factors over time were analysed in detail and compared with equations from the UKPDS outcome model to investigate the need for updating health economic simulation models where the assessment of long-term consequences of risk factors is a key component.

## 2. Material and Methods

### 2.1. The Swedish National Diabetes Register (NDR)

The NDR was established aiming for, inter alia, followup of quality indicators and benchmarking against national guidelines, as has been described elsewhere [22]. Individual-level demographic and clinical data on adults aged ≥18 years who have provided informed consent to participate are reported to the NDR by trained nurses or physicians in all hospital diabetes outpatient clinics and primary health care centres at least once a year. Participation in the NDR is not compulsory. 

### 2.2. Subjects

Altogether 5,043 individuals in the NDR met the general inclusion criteria for this study: (1) type 2 diabetes onset during 2001–2004; (2) 25–70 years old at diagnosis; (3) at least three observations per individual as this is the minimum data requirement for our model; (4) no missing values on smoking or BMI in the year of diagnosis as these were used as covariates in all equations (1449 patients were excluded due to missing values on one or both of these variables). The definition of type 2 diabetes was treatment with diet or oral hypoglycaemic agent (OHA) only regardless the age at onset of diabetes, or treatment with insulin alone or in combination with OHA and age ≥40 years at onset of diabetes. Individuals were included in the analysis if data were available at diagnosis and at two or more measurements after diagnosis for the risk factor under consideration. Sample sizes ranged from *n* = 2,281(LDL cholesterol) to *n* = 4,492 (BMI). While data on HbA1c, systolic BP, and BMI were available in the year 2001, the lipid levels were reported to the NDR starting in 2002. Hence, the sample sizes are smaller in lipid level estimations. Data on risk factor development were available from diabetes diagnosis to the end of 2008. 

### 2.3. Clinical and Demographic Characteristics

Age at diagnosis, gender, duration of diabetes, BMI, smoking, systolic BP, HbA_1c_, TC : HDL, and LDL cholesterol were used in the analyses. The level of HbA_1c_ was measured by the high-performance liquid chromatography (HPLC) Mono-S method following national standards in Sweden. For this study, all HbA_1c_ values were transformed to the Diabetes Control and Complications Trial (DCCT) standard levels using the formula HbA_1c_ (DCCT) = (0.923 × HbA_1c_ [Mono-S]) + 1.345 [23]. Blood pressure recording in the NDR is the mean value of two readings (Korotkoff 1–5) in the supine position according to national guidelines [24]. A smoker was defined as an individual who smoked at least one cigarette per day or used a pipe daily, or who had stopped smoking within the previous 3 months. In cases where there was more than one measurement per year, the yearly mean was used in the analysis.

### 2.4. Statistical Analysis

Keeping the UKPDS outcome model in mind, the time path for risk factors after diagnosis was analysed using a dynamic model where the current level of the risk factor (e.g., HbA_1c_) was allowed to be influenced by its value in the preceding year. Our model included a 1-year lag of the risk factor as follows:

(1) $$\begin{array}{c} \text{R}{\text{F}}_{it}=\alpha \text{R}{\text{F}}_{i,t-1}+\beta_{j}^{\prime }X_{itj}+\varepsilon_{it}, \end{array}$$

(2) $$\begin{array}{c} \varepsilon_{it}=\mu_{i}+\nu_{it}, \end{array}$$

where RF_*it*_ represents the value of the risk facto *r* for *i*th patient (*i* = 1,…, *n*) in year *t* after diagnosis of diabetes (*t* = 1,…, *T*), RF_*i*,*t*−1_ is the 1-year lag of the risk factor, *X*_*itj*_ is a vector of the explanatory variables (*j* = 1,2,…, *J*), and *β* is a vector of coefficients. *μ*_*i*_ is a patient-specific effect which was allowed to vary between patients but had to be constant within patients; *ν*_*it*_ is the identically and independently distributed (i.i.d.) error term with mean zero and variance *σ*_*ν*_. Explanatory variables included the clinical and demographic variables presented above. In addition, to consider the observed decline in HbA_1c_ in the first year after diagnosis, an indicator variable was included in this equation. Moreover, the squares of continuous variables were included in all equations to consider any quadratic relationship. Due to skewness in the variable of duration of diabetes, the log transformation of this variable was used in the model.

As the model in (1) is dynamic, *β* measures the short-term effect of one unit change in the covariate in year *t* on the risk factor. The long-term effect (i.e., change in the risk factor in year *t* and all future years) due to one unit change in the covariate in the year *t* is measured as follows:

(3) $$\begin{array}{c} \frac{\beta }{1-\alpha }. \end{array}$$

Arellano-Bover/Blundell-Bond [25, 26] dynamic panel estimators were used to estimate the risk factor development over up to 7 years after diagnosis of diabetes. Using the Generalized Method of Moments (GMM) developed by Hansen [27], these estimators define the number of lags of the dependent variable, the predetermined variables, and the endogenous variables to be included for the instrument to be valid; and how to combine these valid instruments with the first differences of the strictly exogenous variables. We used xtabond2 command [28] in Stata version 10 for estimating our equations [29]. 

The 5% significance level was considered statistically significant in interpretation of results and we also comment on marginally significant results (*P* < 0.10). The direct effects of each covariate on risk factors were examined individually. In the discussion we return to the total effect of each covariate including also the indirect (simultaneous) effect through BMI. Results are illustrated by 5-year predictions using mean values of covariates for two risk profiles: high risk (smoking man with BMI 32) and low risk (nonsmoking woman with BMI 27).

### 2.5. External Temporal Validation

The results from the time-path equations were used in an external temporal validation [30, 31]. From the NDR, we selected persons aged 25–70 diagnosed with type 2 diabetes during 2005, with risk factor measurement during 2005–2008 (*n* = 414). We first predicted the time path of each risk factor using all covariates significant at the 10% level. We compared these predictions with the observed values up to 3 years after diagnosis. Thereafter, the observed values were regressed on predicted values to test the one-sided hypothesis of positive correlation (H0: *β*1 ≤ 0) [32]. Finally, the performance of our time-path equations relative to those previously reported in the UKPDS outcome model (Table  4 in [13]) was tested by comparing the two sets of predictions with observed values. The model predictions were assessed using the root mean squared error [33].

## 3. Results 

Table 1 shows the clinical characteristics in the year of diagnosis of type 2 diabetes for 5,043 persons in the total sample (column 1) and for 414 persons in the validation sample (column 2). Some characteristics were significantly different between the total sample and the validation sample in the year of diagnosis (Table 1, column 3). 

The median followup was 4 years with 9,536 (LDL) to 25,447 (BMI) person-years of followup data available for the analysis. Although there were missing observations for each risk factor, the results of ANOVA analysis showed that there were no significant differences in the mean of covariates between the available samples and the total sample. 

### 3.1. Time-Path Equations

Table 2 shows the estimated time-path equations. For each equation, the first column shows the short-term effect of one unit change in the covariate while the second column shows the long-term effect of one unit change in the covariate based on (3). 

In all equations, the coefficient of the lagged-dependent variable (the value of dependent variable in previous year) was <1, which implied a convergence of risk factor levels over time, suggesting a decrease in the differences between individuals. Also, the short-term effect captured only part of the changes in covariates and a significant part of the effect became evident in the long term. Generally, a lower age at diagnosis was associated with a higher level of the risk factor, both in the short and in the long term, in all time-path equations except for systolic BP. In addition, higher BMI was correlated to higher values of all other risk factors. 

The interpretation of significant effects for each equation was as follows.

#### 3.1.1. HbA_1c_

The one-unit increase in BMI in the current year raised HbA_1c_ by 0.03% in the long term, after controlling for the other covariates (Table 2, column 1). Higher HbA_1c_ was predicted for people younger at diagnosis and with longer diabetes duration. There was a tendency for women to have lower HbA_1c_ (*P* = 0.08). Smoking was an endogenous covariate indicating that current smoking was affected by previous levels of HbA_1c_, and/or that unobservable factors were correlated to both smoking status and HbA_1c_.

#### 3.1.2. Systolic BP

BMI was positively associated with higher systolic BP and the short- and long-term effects of the one-unit increase in BMI in the current year were 0.02 and 0.03 mmHg, respectively (Table 2, column 2). In the long term, the systolic BP was higher for men (0.12 mmHg) and smokers (0.18 mmHg). 

#### 3.1.3. TC : HDL

Older age at diagnosis was related to a lower TC : HDL (Table 2, column 3). The one-unit increase in BMI in the current year increased the TC : HDL by 0.05 units in the long term. The TC : HDL was lower for females. 

#### 3.1.4. LDL Cholesterol

LDL was lower for people who were older at diagnosis (Table 2, column 4). The level of LDL decreased with duration and increased with BMI, though at a declining rate. The LDL level was higher for females than for males. 

#### 3.1.5. BMI

BMI was higher for people younger at diagnosis. In the long term, BMI was about two units lower for a person who smoked in the current year than for a nonsmoker. BMI increased with diabetes duration. 

### 3.2. Application of the Equations

The results from the time-path equations are illustrated by 5-year predictions for two risk profiles in Table 3. A person with a high-risk profile is a smoking man with BMI 32. A person with a low-risk profile is a nonsmoking woman with BMI 27. Except for BMI, gender and smoking status, the starting values for other risk factors were chosen to reflect the sample mean value of the risk factors in the year of diagnosis (Table 1, column 1). Since a logit model on smoking (not reported; results available on request) did not show any changes in smoking status, it was assumed that the smoking status at baseline is stable over time. Actually, 70% of people who smoked at diagnosis and 95% of people who did not smoke at diagnosis had unchanged smoking status at the last measurement. The predictions in Table 3 include the total effect of covariates on risk factors. We found that although both the person in the low-risk profile (Table 3, top) and the person in the high-risk profile (Table 3, bottom) were predicted to achieve a reduction in four out of five risk factors, the person with the high-risk profile would still experience higher risk levels for most risk factors after 5 years. 

### 3.3. Temporal External Validation

**Figure 1:**
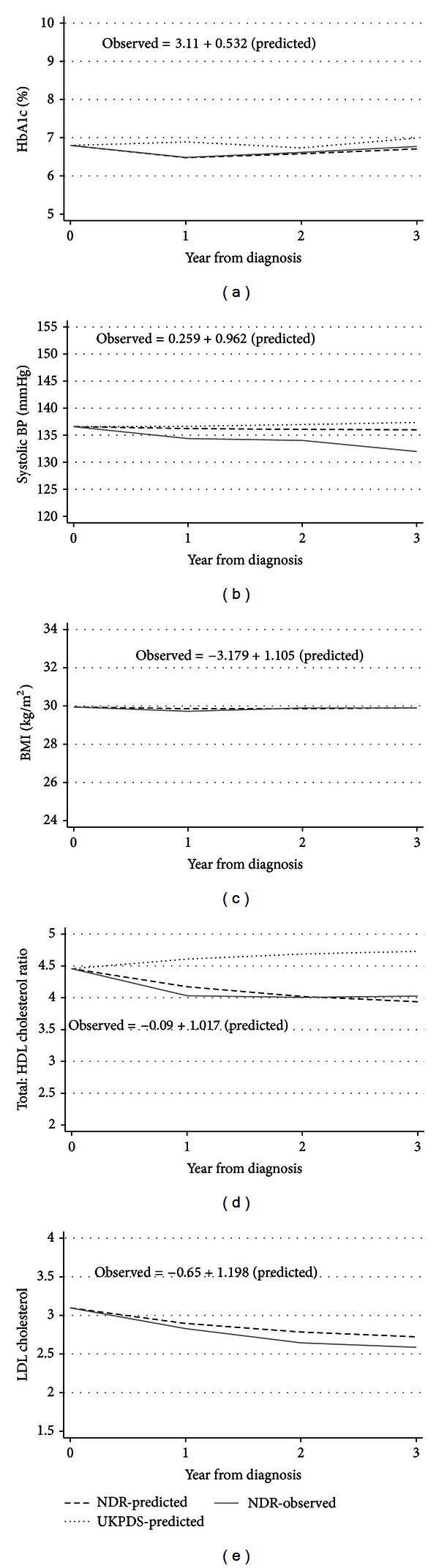
Predicted and observed time paths of risk factors. Time paths of (a) HbA_1c_; (b) systolic BP; (c) BMI; (d) TC : HDL; and (e) LDL cholesterol for nonsmoker males in the validation sample. Equations show the regression of NDR-observed values on NDR-predicted values (BMI and LDL were not estimated in UKPDS outcome model).

A temporal external validation was conducted in four subgroups defined by smoking status and gender from the validation sample consisting of individuals diagnosed with type 2 diabetes in 2005 and registered in the NDR (Table 1, column 2). Figures 1(a)–1(e) show the results of our external temporal validation for nonsmoking males in our validation sample (*n* = 210) (results on smokers and females are available on request). The NDR-predicted values based on the estimated time-path equations in Table 2 (dashed line) corresponded well with the NDR-observed values (solid line), except for systolic BP. The fitted equations in Figures 1(a)–1(e) show the results of regressing observed values on predicted values (positive correlation in all cases; *P* < 0.001). 

The time paths predicted by corresponding UKPDS outcome model time-path equations (Table  4 in [13]; dotted line in Figures 1(a)–1(e)) are clearly different from the predicted and observed NDR values. A comparison between the two models showed that the root mean squared error was lower for our NDR equations than for the UKPDS time-path equations (Table 4). The new time-path equations presented in Table 2 fitted the validation data more closely than the time-path equations previously developed as part of the UKPDS outcome model. 

## 4. Discussion

In this study, the time paths of five major cardiovascular risk factors (HbA_1c_, systolic BP, BMI, TC : HDL, and LDL cholesterol) during 2001–2008 were predicted for individuals diagnosed with type 2 diabetes in the years 2001–2004 in NDR. Using simulation models to predict the development of diabetes-related morbidities and mortalities and their impact on the costs and quality of life is growing in the context of type 2 diabetes. Predicting the changes of risk factors over time and impact of these changes on the progression of complications is a critical part of these models. 

Most outcome analyses and health economic simulation models in diabetes studies are currently based on results obtained from the seminal UKPDS trial. The UKPDS data are based on older cohort, born in the 1920s and 1930s and even earlier, and diabetes management has, partly as a result of the trial, experienced considerable changes since then. It was of particular interest to update previous equations based on newer data on more recent cohorts. The results of temporal external validation showed that except for systolic BP, the NDR-predicted time paths can accurately simulate the actual time path of risk factors for people not used in model development (Figures 1(a)–1(e)). Particularly with regard to HbA_1c_, LDL cholesterol and BMI, the model simulated the observed values fairly well. Compared with the time-path equations in the UKPDS outcome model, our model simulated the time path of risk factors more accurately in Swedish people with type 2 diabetes. 

The value of a risk factor in the preceding year was a significant determinant of the current value of that risk factor. The equations indicated that risk factors converged over time; that is, risk factors approached a certain limit over time. In addition to the short-term effect, changes in covariates in the current year produced effects that lasted longer (long-term effects). The relative effects of short- and long-term effects differed across the different risk factors. In four out of five risk factors, individuals at a higher age at diagnosis had lower values compared with younger individuals. People with higher BMI had higher levels of all other risk factors. 

Using GMM estimation provided some advantages. Firstly, the ordinary least squares (OLS) estimator gives biased results in a dynamic model (also known as “dynamic panel bias”) [34, 35]. Secondly, it was possible to test for endogeneity where covariates were allowed to depend on past values of the dependent variable, but not on future values of it [27, 28]. Thirdly, GMM estimation has been shown to provide consistent results in panels with few time periods and many individuals [28], and works well for unbalanced panels [36]. Moreover, the dynamic specification of the model also enabled us to distinguish between short- and long-term effects of changes in covariates in a single year. 

Our large sample including more than 5,000 patients with type 2 diabetes provided an excellent opportunity to detect the impact of covariates on risk factors. Moreover, compared with clinical trials, using routine clinical practice data for our prediction may increase the generalizability of our results. We also predicted time-path equations for LDL cholesterol and BMI, which were not estimated by previous studies of people with type 2 diabetes. 

Our results are in line with Clarke et al. [13] and Bhargava [37], who also found that the difference between people with different risk levels at diagnosis decreased over time since the coefficients of the lagged dependent variable were <1. In these data, it seems that patients with a higher value of a certain risk factor at diagnosis receive more intensive treatment and subsequently experience a decrease in the level of risk factor. Patients with lower value of certain risk factor seem to experience the inverse trend. 

Lower BMI in smokers has been shown both in the general population and in type 2 diabetes [9, 38]. Other findings have pointed towards a transient relationship between smoking and BMI [39]. Similar to the time-path equations in the UKPDS outcome model [13], we did not find a significant effect of smoking status on most risk factors. Smoking was still correlated to the risk factors through the indirect effect from BMI where smoking was significant.

The negative relationship between age at diagnosis and risk factors implies that younger patients experience a worse risk profile compared with older ones. The result may therefore support more intensive treatment for younger patients. These findings are in line with previous studies. Eliasson et al. [40] reported from Sweden that patients under 50 years of age at diagnosis were considerably more obese and also had higher HbA_1c_ levels.

Being female was generally associated with a lower level of risk factors which is in line with previous findings in Sweden [41] and possibly explain a lower risk of CVD among women with type 2 diabetes compared with men [11, 13]. The gender difference in risk profiles may be caused by differences in genetic factors, behavioural factors (life style, treatment compliance, etc.), or/and treatment modality (choice of treatment, intensity of treatment, etc.). Further studies should explore to what extent these gender differences are modifiable. 

Somewhat worryingly, the higher coefficient on the lagged variable for BMI compared with other risk factors implies that weight loss is less readily achieved among people with type 2 diabetes. Previous studies using NDR data have shown that maintaining a high BMI was associated with higher risk of coronary heart disease (CHD) and CVD [8] and that BMI was an independent predictor of other risk factors in type 2 diabetes [42]. 

Compared with the UKPDS outcome model, our time-path equations produced a lower root mean squared error, implying better predictions in the external temporal validation sample of the Swedish NDR population. However, this does not necessarily imply inaccuracy of any of the estimations. The UKPDS outcome model used patients from a different time period, had different length of followup, applied a different statistical analysis, and used a trial design where patients were selected based on study criteria. There may also be differences in epidemiological features of UK and Swedish type 2 diabetes patients. We furthermore assume that the UKPDS study has affected the treatment for people with type 2 diabetes, producing changes in risk trajectories. We propose that these changes may be the dominating reason for the differences in results illustrated in Figures 1(a)–1(e). Hence, there is a need for equations such as those estimated here that predict time paths of risk factors, and that reflect modern clinical practice. Nevertheless, it would be useful to further test the validity of the two sets of time-path equations using information on risk factors from other diabetes populations or ethnics groups. 

The short followup limited us to consider only the contemporaneous relationships between risk factors and covariates. It was not possible to examine the lagged effect of covariates on current values of risk factors. Hence, examination of the lagged effect of covariates (e.g., BMI and smoking) on current risk factors is an issue for future research. 

### 4.1. Study Limitations

The results of the study should also be interpreted with the study's limitations in mind. Data on smoking may have been affected by selection bias due to underreporting by participants and underrecording by health care staff. We did not control for type of treatment when we examined the effect of patients' characteristics on the time path of risk factors. Treatment would have been an endogenous covariate in our equations as it is typically determined by the level of risk factors. This strategy has been adopted also in previous studies [13, 37]. The definition of type 2 diabetes used in this study excluded most patients with type 1 diabetes, as <1% were aged <30 years at diagnosis, and <5% had an age at diagnosis of <40 years in different equations. There were no exclusion criteria regarding presence or absence of co-morbidities for participation in this study (in contrast to randomized clinical trials), which may have decreased the precision of the results. However, this reflects the routine practice across health centres in Sweden. The equations were developed based on data in Swedish population and this may limit the generalizability of results to other populations. 

## 5. Conclusion

In sum, the predictions of changes in risk factors over time are needed for informed decision making by clinicians and policy makers. As first step in developing an outcome model for type 2 diabetes in Sweden, the time paths of five major risk factors were predicted here. The results showed the importance of updating the equations as new data become available. The equations presented here can be used by anyone interested in predicting the future level of risk factors using population characteristics of relevance to their specific decision problem. Moreover, using these updated equations in constructing simulation models in health economic studies may result in more accurate models for evaluating alternative treatment strategies for type 2 diabetes. 

## Figures and Tables

**Table 1:** Total and validation samples characteristics in the year of diagnosis of diabetes.

Variable	Total sample	Validation sample	No difference
	(*n* = 5043)	(*n* = 414)	*P* value
	Mean (±SD)	Mean (±SD)	
	(1)^a^	(2)	(3)
Male (*n*)	2967	244	
Age at diagnosis	56.0 (8.8)	57.0 (8.0)	0.11
HbA_1c_ (%)	7.0 (1.4)	6.8 (1.2)	0.02
BMI (Kg/m^2^)	29.8 (4.9)	29.8 (4.7)	0.87
Systolic BP (mmHg)	138.5 (17.9)	135.9 (16.6)	0.03
TC : HDL^b^	4.6 (1.4)	4.5 (1.2)	0.12
LDL cholesterol (mmolL^−1^)^b^	3.1 (1.0)	3.1 (1.0)	0.98
Smokers (proportion, %)	21.0	14.0	<0.01
Female (*n*)	2076	170	
Age at diagnosis	57.0 (9.0)	59.0 (7.0)	<0.01
HbA_1c_ (%)	6.9 (1.3)	6.8 (1.1)	0.07
BMI (Kg/m^2^)	30.7 (5.9)	30.4 (5.7)	0.42
Systolic BP (mmHg)	138.9 (18.2)	138.8 (15.6)	0.97
TC : HDL	4.4 (1.4)	4.2 (1.4)	0.19
LDL cholesterol (mmolL^−1^)	3.3 (1.0)	3.2 (1.0)	0.75
Smokers (proportion, %)	22.0	18.0	0.23

^a^The *P* values based on ANOVA analysis showed that there were no statistically significant differences in the means of risk factors between total sample and estimation subsamples for time paths of risk factors. ^b^As data on lipid levels were collected since 2002, these figures are based on data for 3214 patients.

**Table 2:** Estimates of GMM for risk factors from NDR data.

	(1)		(2)		(3)		(4)		(5)	
Risk factors	HbA_1c_^b^		Systolic BP^c ^ (mmHg)		TC : HDL		LDL (mmolL^−1^)		BMI (Kg/m^2^)	
	Coefficient	Long-term effect	Coefficient	Long-term effect	Coefficient	Long-term effect	Coefficient	Long-term effect	Coefficient	Long-term effect
Constant	3.055***	—	5.546**	—	1.647**	—	1.938***	—	6.832*	—
Ln (diabetes duration)	0.182***	0.384	-0.050	−0.094	0.099	0.216	−0.059***	−0.091	0.106***	0.561
Year 1^a^	−0.144*	−0.304	NA	—	NA	—	NA	—	NA	—
Age at diagnosis	−0.008***	−0.017	0.021*	0.039	−0.009**	−0.020	−0.007***	−0.011	−0.022**	−0.116
Female	−0.034*	−0.072	−0.064**	−0.120	−0.144***	−0.314	0.060***	0.093	0.134	0.709
Smoking	0.192	0.405	0.094***	0.176	0.071	0.155	0.091	0.140	−0.370***	1.958
BMI	0.015***	0.032	0.017***	0.032	0.021***	0.046	0.018**	d	—	—
BMIsquared	NS	—	NS	—	NS	—	−0.0003**	d	—	—
Lag of HbA_1c_	0.526***	—	NA	—	NA	—	NA	—	—	—
Lag of SBP	NA^e^	—	0.466**	—	NA	—	NA	—	—	—
Lag of TC : HDL	NA	—	NA	—	0.541***	—	NA	—	—	—
Lag of LDL	NA	—	NA	—	NA	—	0.352***	—	—	—
Lag of BMI	NA	—	NA	—	NA	—	NA	—	0.811***	—

*N* (patients)	4450		4158		2274		2068		4284	
Person years	20699		20144		10157		9536		25447	
Hansen test	0.053		0.588		0.654		0.461		0.126	
AR1^f^	<0.001		<0.001		<0.001		<0.001		<0.001	
AR2	0.356		0.095		0.219		0.310		0.078	

^∗∗∗,∗∗,∗^Denote significance level at the 1, 5, and 10%, respectively; Hansen test on overidentifying restrictions; AR1 and AR2 show the test on first and second order autocorrelation, respectively. ^a^Year 1: 1 if diabetes duration = 1 year, 0 otherwise. ^b^In HbA_1c_ equation, the smoking was an endogenous variable. ^c^Systolic BP values transformed as systolic BP/10. ^d^As the BMI squared is significant, the long-term effect will be different for different levels of BMI. ^e^The covariate was not included in the estimation. ^f^The strong evidence against null hypothesis of no autocorrelation in the first-differenced errors is expected in the model.

**Table 3:** Prediction of risk factors for two hypothetical patients over 5 years after the diagnosis.

Risk factor	Value in year of diagnosis	Year 1	Year 2	Year 3	Year 4	Year 5
	Patient 1: woman with BMI equal to 27 and 60 years old in the year of diagnosis and nonsmoking					

HbA_1c_ (%)	7.00	6.49	6.50	6.58	6.68	6.78
Systolic BP (mmHg)	138.00	136.43	135.76	135.52	135.46	135.48
BMI (Kg/m^2^)	27.00	27.41	27.81	28.18	28.51	28.80
TC : HDL	4.50	3.98	3.70	3.56	3.49	3.46
LDL cholesterol (mmolL^−1^)	3.00	2.90	2.83	2.78	2.75	2.72

	Patient 2: man with BMI equal to 32 and 60 years old in the year of diagnosis and smoking at diagnosis					

HbA_1c_ (%)	7.00	6.58	6.62	6.71	6.80	6.88
Systolic BP (mmHg)	138.00	138.63	138.82	138.82	138.75	138.68
BMI (Kg/m^2^)	32.00	31.09	30.43	29.93	29.56	29.29
TC : HDL	4.50	4.20	4.02	3.91	3.85	3.81
LDL cholesterol (mmolL^−1^)	3.00	2.85	2.75	2.69	2.66	2.63

**Table 4:** Root mean squared error of the regression of observed on predicted values in nonsmoker males.

Risk factor	Root mean squared error	
	NDR equations	UKPDS outcome model
HbA_1c_	12.60	15.23
Systolic BP	18.96	20.15
TC : HDL	13.72	16.13
